# Evaluating Electrolyte–Anode Interface Stability in Sodium All-Solid-State Batteries

**DOI:** 10.1021/acsami.2c12759

**Published:** 2022-10-14

**Authors:** Grayson Deysher, Yu-Ting Chen, Baharak Sayahpour, Sharon Wan-Hsuan Lin, So-Yeon Ham, Phillip Ridley, Ashley Cronk, Erik A. Wu, Darren H. S. Tan, Jean-Marie Doux, Jin An Sam Oh, Jihyun Jang, Long Hoang Bao Nguyen, Ying Shirley Meng

**Affiliations:** † Program of Materials Science and Engineering, University of California San Diego, La Jolla, California92093, United States; ‡ Department of NanoEngineering, University of California San Diego, La Jolla, California92093, United States; § Pritzker School of Molecular Engineering, The University of Chicago, Chicago, Illinois60637, United States

**Keywords:** anode−electrolyte interface, solid electrolyte, sodium, chloride, sulfide, borohydride

## Abstract

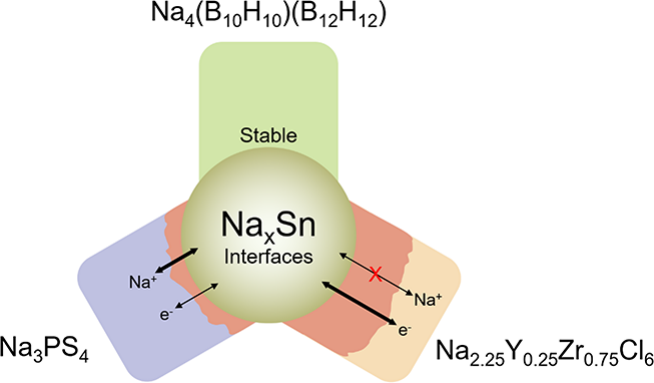

All-solid-state batteries have recently gained considerable attention due to their potential improvements in safety, energy density, and cycle-life compared to conventional liquid electrolyte batteries. Sodium all-solid-state batteries also offer the potential to eliminate costly materials containing lithium, nickel, and cobalt, making them ideal for emerging grid energy storage applications. However, significant work is required to understand the persisting limitations and long-term cyclability of Na all-solid-state-based batteries. In this work, we demonstrate the importance of careful solid electrolyte selection for use against an alloy anode in Na all-solid-state batteries. Three emerging solid electrolyte material classes were chosen for this study: the chloride Na_2.25_Y_0.25_Zr_0.75_Cl_6_, sulfide Na_3_PS_4_, and borohydride Na_2_(B_10_H_10_)_0.5_(B_12_H_12_)_0.5_. Focused ion beam scanning electron microscopy (FIB-SEM) imaging, X-ray photoelectron spectroscopy (XPS), and electrochemical impedance spectroscopy (EIS) were utilized to characterize the evolution of the anode–electrolyte interface upon electrochemical cycling. The obtained results revealed that the interface stability is determined by both the intrinsic electrochemical stability of the solid electrolyte and the passivating properties of the formed interfacial products. With appropriate material selection for stability at the respective anode and cathode interfaces, stable cycling performance can be achieved for Na all-solid-state batteries.

## Introduction

To accommodate the rapidly growing adoption rates of renewable energy sources, such as wind and solar, grid energy storage systems are a complementary and required technology to store the TWh levels of energy produced and consumed each day.^1,2^ Currently, grid energy storage technologies include hydro pumps, compressed air, flywheels, and secondary batteries.^3^ Na battery technologies recently emerged as a prospective candidate for grid storage applications, thanks to ubiquitous Na material sources and a lower overall cost per kWh.^4−7^ Although the gravimetric energy densities of Na batteries are inherently lower than their Li counterparts, they can still potentially achieve competitive volumetric energy densities, making them suitable for stationary applications. Beyond energy densities, safety is also a metric of paramount importance especially when introducing large amounts of batteries into urban and densely populated environments. One promising approach to address this safety requirement is to replace the flammable liquid electrolyte in conventional batteries with a nonflammable solid-state electrolyte (SSE).^8^ To this end, Na all-solid-state batteries (Na-ASSBs) have the potential to be a cost-effective system for future grid storage applications with superior safety factors in mind.

Alloy-based anodes, such as Sn, offer high specific capacities (847 mAh g^–1^) and are prime candidates to serve as high-energy anodes for Na-ASSBs, offering the potential to reach volumetric energy densities that meet or exceed that of commercial lithium-ion batteries.^9^ Nonetheless, Sn anodes exhibit low reduction potentials (∼0.1 V vs Na/Na^+^), and thus, it is important to select and design solid electrolytes with good reduction stability to serve as the anolyte to ensure stable long-term cycling. Extensive exploration has led to the discovery of several SSE materials with Na^+^ conductivities comparable to many liquid electrolytes at ambient temperature.^10−15^ Oxide SSEs have previously been explored due to their wide electrochemical stability windows and high ionic conductivities.^16,17^ However, due to significant grain boundary resistances, which require high-temperature sintering to mitigate, their application in Na-ASSBs remains limited.^18^ Sulfide-based SSEs have been introduced to overcome the need for high-temperature processability, while retaining high ionic conductivities, and have recently been shown to work well in polymer composites to further improve the processability of the separator layers.^19−21^ Additionally, chloride-based materials have been explored due to their tolerance to highly oxidative potentials (up to ∼4.2 V vs Na/Na^+^) and high ionic conductivity, along with practical room temperature processability.^16,22^ Recently, sodium borohydride SSEs have been introduced as some of the fastest Na^+^ conductors at room temperature.^23−25^ Yet, it remains unclear how stable, if at all, these SSE materials are when in direct contact with the anode.

In a solid-state battery, interphase layers can be formed at the electrode–electrolyte interface due to chemical and electrochemical instabilities between the materials.^26−28^ The formation of an interlayer can potentially slow down the Na^+^ diffusion process and reduce the battery capacity due to the loss of reversible Na^+^ inventory.^22^ Interphase layers can be classified into three categories: (i) ionically insulating and electronically conducting, (ii) ionically conducting and electronically conducting, and (iii) ionically conducting and electronically insulating. Any electronically conductive interlayer will not passivate and will continue to grow after each (dis)charging cycle, irreversibly consuming Na^+^ inventory. The most commonly observed interlayer species are type (ii), which is also referred to as a mixed-conducting interface (MCI). This has been demonstrated previously for electrolytes such as the highly conductive Na_3_SbS_4_, in which Sb is reduced to Na–Sb alloy (an ionic and electronic conductor), resulting in the continual growth of the MCI.^29^ Consequently, a cell using Na_3_SbS_4_ against Na anode exhibited significant capacity fade during cycling. This phenomenon was also reported in lithium-based solid electrolyte materials such as Li_10_GeP_2_S_12_, forming an electronically conductive interphase containing Li_*x*_Ge that results in significant capacity fade during cycling.^30,31^ On the other hand, if the interfacial degradation products are ionic conductors and electronic insulators, then a passivating solid electrolyte interface (SEI) is formed, preventing subsequent degradation.

Previously, anodic stability of Na-SSEs has typically been evaluated using a symmetric cell architecture in which the SSE of interest is sandwiched between two Na-containing anodes such as Na_*x*_Sn.^22^ Then, Na is shuttled back and forth between the electrodes while recording the potential response during numerous cycles. It has previously been assumed that if the potential response remains stable during later cycles, then the SSE is stable. However, this is not always the case, as we will demonstrate in this work. The origin of this discrepancy is the nature of the formed interphase layer.

Due to the variable nature of the formed interphase layer, symmetric cell cycling is not always an appropriate method for evaluating SSE stability. For example, if the interphase layer is a fast ion conductor, then there will be minimal change to the cell polarization, even though there could be significant growth in the interlayer thickness and consumption of active sodium. Instead of using a symmetric cell architecture to evaluate SSE stability, the instability of an SSE should be quantified using a full-cell architecture with a limited amount of sodium. The amount of irreversible Na will provide more useful quantitative insight into the severity of SSE (in)stability that will be needed to properly assess an SSE candidate’s suitability for use in Na-ASSB full cells with limited Na.

In this study, we evaluated the electrode–electrolyte interface of three SSE candidates against a representative (Na)Sn alloy anode: sulfide-based Na_3_PS_4_ (NPS), chloride-based Na_2.25_Y_0.25_Zr_0.75_Cl_6_ (NYZC), and borohydride-based Na_2_(B_10_H_10_)_0.5_(B_12_H_12_)_0.5_ (NBH). These three candidates represent the most commonly used Na-SSE families in recent literature. The formation and evolution of the SSE–(Na)Sn interphases were characterized by focused ion beam scanning electron microscopy (FIB-SEM) coupled with energy-dispersive X-ray spectroscopy (EDS) for elemental mapping, X-ray photoelectron spectroscopy (XPS), and electrochemical impedance spectroscopy (EIS). The obtained results revealed that NYZC is incompatible with the (Na)Sn anode due to the formation of an electronically conductive interlayer, leading to a rapid cell failure from the first cycle. The sulfide NPS exhibited the formation of an MCI, but due to a relatively lower electronic conductivity of its reduced products, there was less severe interlayer formation compared to NYZC. In contrast, a stable interface, and thus stable electrochemical cycling, was achieved when utilizing the NBH electrolyte, which did not form any detectable interlayer against the (Na)Sn anode.

## Results

### Initial Evaluation in Full-Cell Configuration

**Figure 1:**
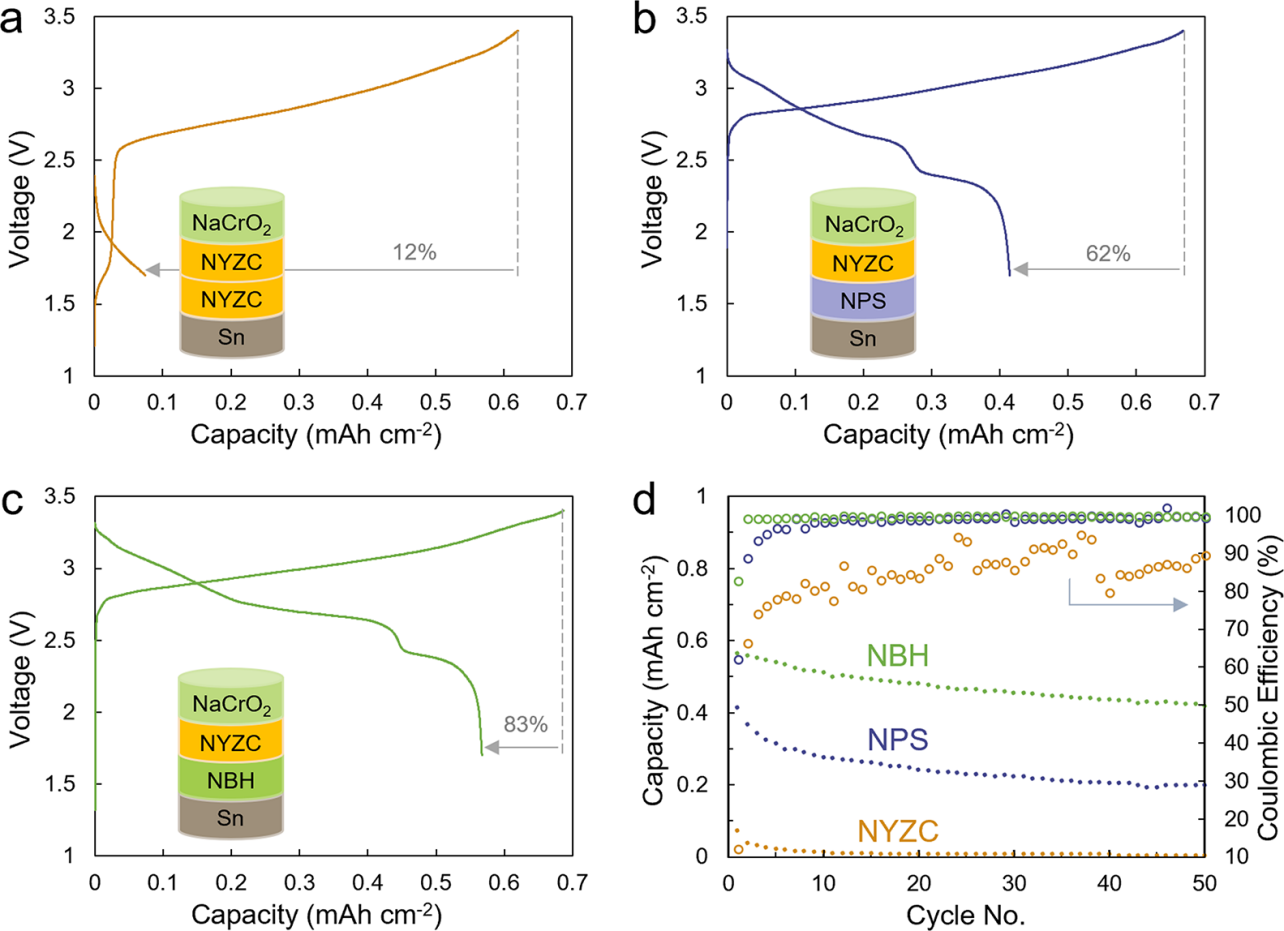
First cycle voltage curves of Sn | SSE | NYZC | cathode composite cells using (a) NYZC, (b) NPS, and (c) NBH electrolytes. (d) Extended cycling data for the same three full cells. The cells were cycled at 0.064 mA cm^–2^ (C/10).

Three Sn | SSE | NaCrO_2_ composite full cells were assembled (SSE: NYZC, NPS, or NBH). The full-cell system with a fixed amount of sodium was used to better understand the rate of Na inventory losses. In all cases, NYZC was used at the catholyte due to its oxidative stability (Figure S1), along with an additional layer between the separator and the cathode to prevent direct contact between the cathode and the SSE of interest. The capacity ratio between the negative and positive electrodes (N/P ratio) was set at 1.2 for all three cells. The cell using NYZC as the separator showed rapid and irreversible failure, with a low initial Coulombic efficiency (ICE) of 12% (Figure 1a); its capacity faded rapidly to a negligible value after 10 cycles. The cell using NPS showed a higher ICE of 62%, but a significant capacity fade was still observed over the first 10 cycles (Figure 1b). Among the three candidates, NBH showed the best ICE of 83% and capacity retention (Figure 1c). Interestingly, all three cells displayed a similar first charge capacity, indicating successful desodiation of the cathode. However, the cell discharge capacities exhibited stark differences. With the same Na inventory sent to the Sn anode in all three cells, the varying rates of Na inventory losses indicate that irreversible losses must therefore occur at the SSE–Sn interface. As the anode was pure Sn without any anolyte mixed in (i.e., not a composite anode), any interfacial degradation should occur at the contact plane between the SSE and the Sn electrode. To characterize this interphase formation, half cells using excess Na reservoir from a Na_9_Sn_4_ counter electrode were used. Excess Na allows higher capacity cycling that should amplify the effects of any interface reactions, enabling for better detection and analysis.

### SSE–Sn Interface Investigation

**Figure 2:**
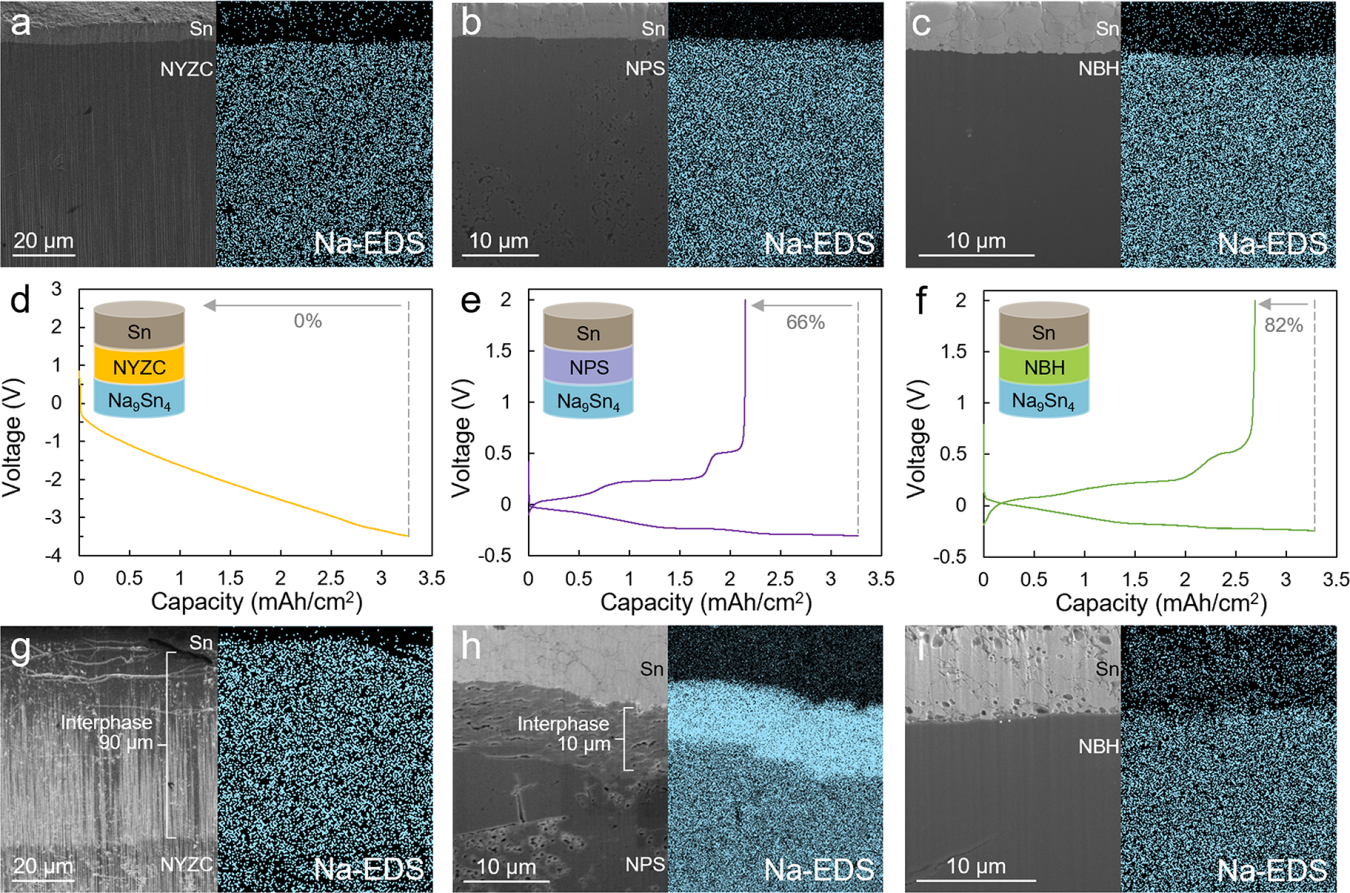
Cross sections and Na-EDS mapping of the Sn|SSE interface of uncycled cells using (a) NYZC, (b) NPS, and (c) NBH electrolytes. Each cell was cycled once using 0.16 mA cm^–2^, and the cycling data for (d) NYZC, (e) NPS, and (f) NBH are shown. Cross sections and Na-EDS mapping of the Sn|SSE interface of the cells using (g) NYZC, (h) NPS, and (i) NBH electrolytes after cycling.

To examine the SSE–Sn interface, three half cells (Na_9_Sn_4_ | SSE | Sn) were constructed. After 24 h of contact, FIB milling was used to cut through the Sn and electrolyte layers to expose the 2D interface. In the pristine state, dense Sn and SSE layers were observed in all cells; no interface layer was observed for any of the electrolytes based on SEM imaging and Na elemental mapping (Figure 2a–c). This suggests that the pristine Sn electrode is chemically stable with the electrolytes. To further verify their chemically stable nature, Sn powder was mixed with each of the SSEs and heated for 10 h at 80 °C. After heating, XRD results (Figure S2) showed that Sn and the pristine SSE remained intact. This suggests that even under harsh conditions, Sn is chemically stable with NYZC, NPS, and NBH. Besides chemical stability, electrochemical stability is the other important aspect to evaluate.

To probe the electrochemical stability of the SSEs with the (Na)Sn anode, the three half cells (Na_9_Sn_4_ | SSE | Sn) were cycled with the same Na capacity shuttling to the Sn electrode using a capacity cutoff protocol. The sodiated Na_*x*_Sn was then desodiated to a 2.0 V voltage cutoff (Figure 2d–f). Similar to the full-cell cycling results (Figure 1), the same trend in Coulombic efficiency was observed for these half cells. NYZC was not able to cycle beyond the first Sn sodiation step, and the overpotential of the cell quickly approached 4 V, indicating a significant increase in the cell impedance. On the contrary, the NPS cell exhibited a higher efficiency (66%), while NBH exhibited a highest efficiency of 82%.

FIB milling was conducted after one cycle to probe the evolution of the SSE–Sn interface (Figure 2g–i). The interphase layers were characterized using EDS mapping to probe the Na concentration gradients to distinguish the SSE, Sn, and interphase layers. Any interlayer formed as a result of the reduction of the SSE would contain a higher concentration of Na compared to the pristine SSE. A 90 μm-thick interphase layer, highlighted by a high Na content, was observed in the cell with NYZC separator (Figure 2g). This finding correlates well with the observed cycling behavior, as such a thick interphase layer would be expected to cause a steep increase in the cell impedance and polarization (Figure 2d). For the NPS cell, a 10 μm-thick interphase layer was observed. Such a thinner interphase layer would result in less irreversible Na inventory consumption and a higher ICE (Figure 2e) compared to NYZC. Contrary to NYZC and NPS, no interlayer was observed for the NBH cell; no noticeable change in the elemental mapping was seen at this interface (Figure S3).

**Figure 3:**
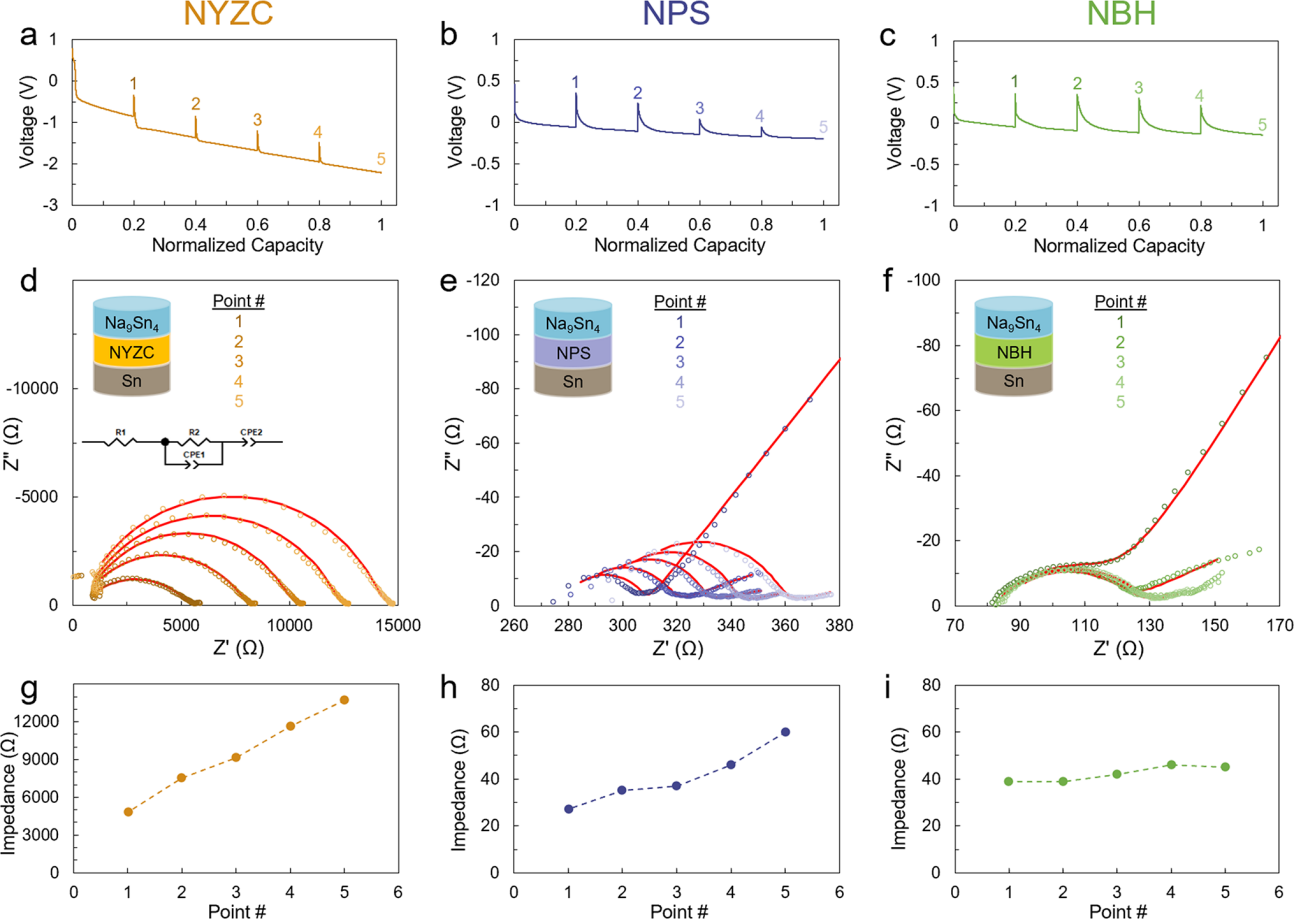
Voltage curves of Na_9_Sn_4_ | SSE | Sn half cells using (a) NYZC, (b) NPS, and (c) NBH electrolytes cycled at 0.16 mA cm^–2^. Impedance growth during Sn sodiation for the Na_9_Sn_4_ | SSE | Sn half cells using (d) NYZC, (e) NPS, and (f) NBH electrolytes. Interfacial impedance growth during sodiation for (g) NYZC, (h) NPS, and (i) NBH based on the EIS fitting results.

To further evaluate and quantify the effect of the formed interphase layers on cell impedance, EIS measurements were performed using the Na_9_Sn_4_ | SSE | Sn half-cell configuration, where the same areal capacity of Na (1.4 mAh cm^–2^) was sent to the Sn electrode for all three cells (Figure 3). EIS measurements were conducted at five different intervals during the sodiation process to track any impedance growth (Figure 3a–c). Nyquist plots for NYZC, NPS, and NBH cells (Figure 3d–f) were fitted using the equivalent circuit shown in Figure 3d, and the interface resistance values are plotted in Figure 3g–i. For NYZC, the interfacial resistance increased by ∼9000 Ω during the Sn sodiation, which agrees with the high polarization of the cell (Figure 3a). The interfacial degradation in NPS was less severe than NYZC as evidenced by a slight increase (∼35 Ω) in the interfacial resistance (Figure 3b,h). On the other hand, the NBH cell exhibited negligible change in the interfacial resistance, implying the absence of any significant resistive layer at the Sn–NBH interface (Figure 2i).

### SSE–Sn Interface Composition Analysis

**Figure 4:**
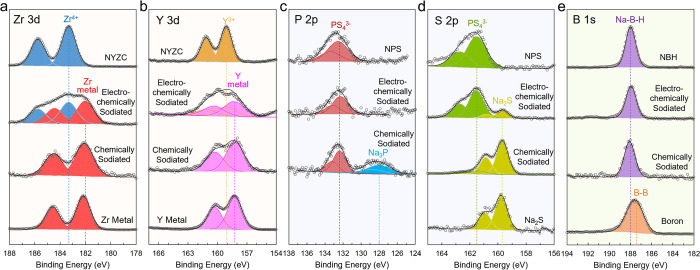
(a) Zr 3d, (b) Y 3d, (c) P 2p, (d) S 2p, and (e) B 1s XPS spectra for NYZC, NPS, NBH, and the electrochemically and chemically sodiated SSEs. Zr metal, Y metal, Na_2_S, and B are also added as references.

XPS was employed to identify the chemical composition of degradation products formed at the SSE–Sn interface (Figure 4). Three sets of samples were produced for each SSE: (i) pristine electrolytes as references, (ii) electrochemically sodiated samples recovered from the electrochemical cycling of Na_9_Sn_4_ | SSE | Sn half cells, and (iii) chemically sodiated samples obtained by directly mixing SSEs with Na metal. In the fabrication of electrochemically sodiated samples, extra SSE was intentionally added to the Sn electrode in the Na_9_Sn_4_ | SSE | Sn half cells to increase the contact area and amplify XPS signals corresponding to the degradation products. All the chemically sodiated samples were subjected to XRD measurements for identification purposes.

**Figure 5:**
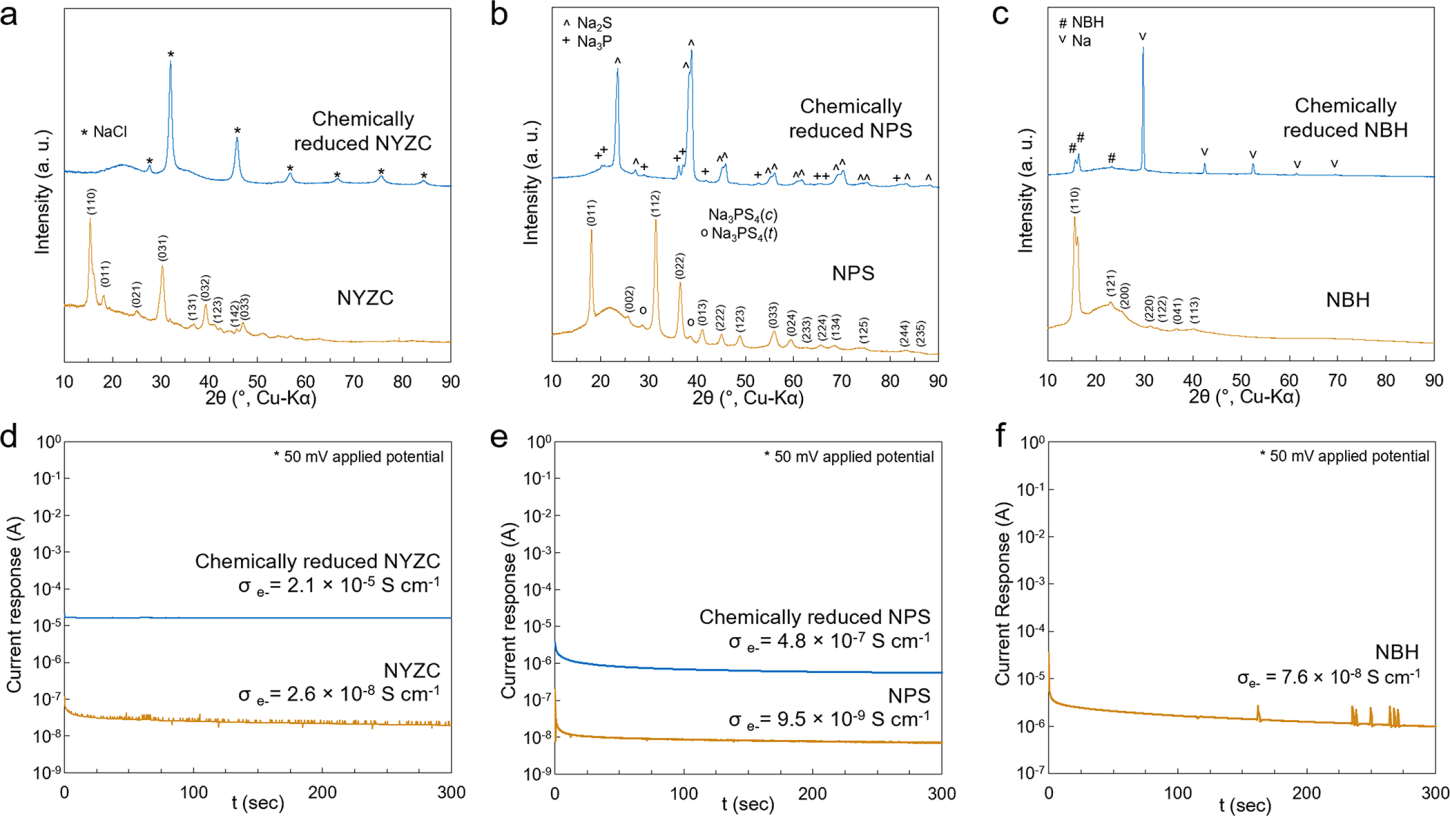
XRD of (a) NYZC, (b) NPS, and (c) NBH after mixing with Na metal and DC polarization electronic conductivity measurements of the reduced (d) NYZC and (e) NPS interphase materials along with (f) pristine NBH.

Zr and Y metals were detected in the electrochemically sodiated NYZC as evidenced by the emergence of a new set of peaks at lower binding energies (Figure 4a,b). Peaks corresponding to pristine NYZC are still present in the electrochemically sodiated sample as part of the NYZC in the composite might not be in direct contact with Sn particles. When chemically sodiated, the XPS signatures of pristine NYZC completely disappeared, and only Zr and Y metal peaks were observed. The XRD pattern of chemically sodiated NYZC only shows the diffraction peaks of NaCl, supporting a complete consumption of NYZC when it was in contact with Na metal (Figure 5a). The lack of any Bragg peaks associated with Y and Zr metals can be explained by the amorphization or the formation of nanocrystals that has been known to occur during the electrochemical cycling of some conversion-type electrode materials.^32−34^ The electrochemical reduction of NYZC to Zr and Y to their metallic state is in agreement with reported computational work.^22^ The reduction of Zr or Y to the metallic state has also previously been observed in Li solid electrolytes when Li_7_La_3_Zr_2_O_12_ or Li_3_YCl_6_ was in contact with Li metal, respectively.^35,36^ The reduction reaction of NYZC can be written as follows:
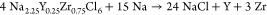
1

When electrochemically sodiated, the NPS sample showed a new set of peaks in the S 2p region corresponding to Na_2_S (Figure 4c). The presence of Na_2_S is even more pronounced in the chemically sodiated one (Figure 4d). New signals are observed in the P 2p region of chemically sodiated NPS and are assigned to the presence of Na_3_P based on previous computational predictions.^29,37^ The presence of Na_2_S and Na_3_P in chemically sodiated NPS was also confirmed by the use of XRD (Figure 5b), which is in agreement with previous work of Wenzel *et al*.^38^ The reduction of NPS into Na_2_S and Na_3_P is summarized in eq 2:
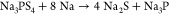
2

The NBH electrolyte showed no noticeable change in the B 1s peak position (188.0 eV) for neither the electrochemically nor the chemically sodiated samples (Figure 4e). The boron (B^0^) reference exhibits a peak at lower binding energy (187.5 eV), thus indicating that the B in NBH was not reduced to its neutral state. Moreover, the XRD pattern recorded on the chemically sodiated NBH only shows the diffraction peaks of pristine NBH and Na metal (Figure 5c), demonstrating the stability of NBH in strong reductive conditions. The NBH electrolyte contains two different borohydride anions, [B_10_H_10_]^2–^ and [B_12_H_12_]^2–^, whose structures are constructed based on the covalent heccaidecadeltahedron B_10_ and icosahedron B_12_, respectively. Each boron vertex is covalently connected to a hydrogen atom, and the negative charge is delocalized over the [B_*x*_H_*x*_]^2–^ cages.^39^ The hydrogens in [B_*x*_H_*x*_]^2–^ are hydride, bearing a negative charge, which cannot be further reduced. Moreover, the bonding between boron atoms in the B_10_ and B_12_ polyhedra is highly covalent in nature, which cannot be easily broken by any reducing agents. A combination of these two factors offers NBH a significant resistance to reduction even at 0 V vs Na/Na^+^.^40−42^

## Discussion

The electrochemical cycling of Na_9_Sn_4_ | SSE | Sn half cells using NYZC, NPS, and NBH as separators exhibited significantly different behaviors (Figure 2d–f). The cell with NYZC showed instant failure from the first cycle, while NPS and NBH exhibited a higher ICE of 66 and 82%, respectively. Furthermore, an interphase layer of 90 and 10 μm was observed in the cells using NYZC and NPS as separators, while none was detected with NBH (Figure 2g–i). As the degree of interface passivation depends greatly on the electronic property of the degradation products within the interlayer, DC polarization was performed on the chemically sodiated SSEs to determine their electronic conductivity (Figure 5d).

The reduced NYZC sample exhibited an increase in electronic conductivity by three orders of magnitude compared to the pristine material, which was assigned to the formation of Y and Zr metals (eq 1 and Figure 4a,b), which are good electron conductors.^43^ The formation of an electron-conducting layer at the NYZC–Sn interphase induces a rapid and continuous consumption of NYZC and a growth in interlayer thickness (Figure 2g). NaCl was also detected as a degradation product of chemically sodiated NYZC (Figure 5a); however, the ionic conductivity of NaCl is almost negligible at ambient temperature (σ_Na^+^_ = 10^–15^ S cm^–1^),^44^ leading to a significant increase in the cell polarization (Figure 3d). NYZC represents the worst-case scenario for an interlayer at the anode side in ASSBs, where the degradation products are electronically conductive and ionically insulating, resulting in a continuous consumption of Na inventory and an excessively high interface impedance. Therefore, this class of SSEs cannot be used with low-voltage anodes even if there is a large excess of Na reservoir in the system.

DC polarization measurements on chemically sodiated NPS showed an increase in electronic conductivity by about two orders of magnitude compared to the pristine material (Figure 5e). As Na_2_S in the interlayer is an electronic insulator, the increase in electronic conductivity can be attributed to Na_3_P, which was predicted to have a narrow band gap of 0.5 eV.^45^ This electronic conductivity increase paired with the known fast Na^+^ diffusion in Na_3_P^46^ indicates that the reduction of NPS results in the formation of an MCI layer, in which Na_3_P is the main electronic and ionic conductor. Intuitively, NPS is a prime example of why galvanostatic cycling of symmetric cells is not always a reliable method for evaluating interface stability. As shown in Figure S4, the polarization of the cell during plating/stripping remained relatively constant. This would suggest that the interface is stable. However, based on our results presented here, it is known that the interface is not stable. We attribute this counterintuitive result to the mixed-conducting interlayer present after the reduction of NPS. Due to the ionically conductive interlayer, there is minimal change to the cell polarization. In contrast, the interlayer formed from the reduction of NYZC is ionically insulating, resulting in significant cell polarization during plating/stripping cycles (Figure S4). Furthermore, the interfacial degradation with NPS is less severe than NYZC (Figures 2h and 3e), which can be explained by the lower electronic conductivity of Na_3_P compared to Zr and Y metals. While NPS is commonly used in Na-ASSBs,^22,47^ it has always been paired with anodes containing excess Na. Any Na consumed in the MCI formation can be compensated by a nearly unlimited Na reservoir in the anode, which explains the apparent high-capacity retention of reported cells. Based on the data presented here, the use of NPS should be carefully evaluated, particularly when designing commercial full cells in which the separator thickness is reduced to 10–25 μm and the amount of Na in the system is limited.

NBH exhibited superior stability against reduction conditions, and no degradation products could be detected in the conditions tested here (Figure 5c). In an electrochemical cell, the NBH–Sn interface is electrochemically stable or at least is passivated by an SEI layer with a negligible thickness (Figures 2i and 3i), allowing for the observed ∼99% Coulombic efficiency over long-term cycling (Figure 1d). The reduction stability of NBH is a synergistic effect of the utilization of the hydride anion (H^–^) and highly stable boron clusters.

The data presented here highlights the importance of utilizing suitable SSEs to achieve a stable SSE–anode interface to ensure a stable long-term cycling in Na-ASSBs. No anolyte was utilized in all the cell architectures in this study, and thus, the interfacial degradation was limited to the 2D contact between the Sn anode and the SSE separator, which is effectively the least severe scenario. In a practical cell design, a composite anode with a higher contact area between SSEs and Sn may be employed, and any interfacial degradation will be intensified or accelerated. Nonetheless, the information accumulated in this work points out some important future directions for SSE selection and design to achieve a stable SSE–anode interface; as the SSE is subjected to reductive potentials when it is in direct contact with the anode, an ideal SSE at the SSE–anode interface should be completely stable against all reduction conditions or at least able to form an ionically conductive and electronically insulating passivation layer.

Beyond the first few cycles in which the interlayers are expected to form, some capacity fade was still observed during later cycles using the NPS or NBH separator. FIB milling was conducted on the cells after 1000 cycles (Figure S5). The morphology of the Sn and interphase layers in the Sn | NPS | NYZC | NaCrO_2_ cells was similar to that observed in the half cell (Figure 2h) in which an interphase layer was observed at the interface between the Sn anode and the NPS electrolyte. Interestingly, the cell using NBH still did not exhibit any noticeable interphase layer even after many cycles (Figure S5b). This reiterates the highly stable nature of the NBH material against the reductive environment at the Na_*x*_Sn electrode. Sn pulverization was observed in the NBH cell as evidenced by the disconnected Sn particles compared to the dense pristine Sn layer (Figure 2c). This was likely the origin of the observed slow capacity fade, as a loss of electronic contact between the Sn particles and current collector would result in those Sn particles becoming inactive. Sn pulverization can be attributed to the significant amount of volume change that occurs during the (de)sodiation of Sn (420%).^48^ Due to the consumption of Na inventory when forming the interphase layers with NYZC and NPS electrolytes, the Sn received less Na and therefore likely did not undergo as much volume change compared to the NBH cell, which underwent two times more exchanged capacity compared to the NPS during the course of the study. Therefore, a dense Sn layer was still observed after long cycling with NPS as the electrolyte (Figure S5a). This data suggests that the main mechanism for capacity fade when using NPS is interlayer formation and consumption of sodium inventory. This can be seen both visually in the cross-sectional imaging and in the Coulombic efficiency data. The low ICE followed by several more cycles of <99% efficiency is the typical signature of interlayer growth and subsequent passivation. Conversely, the main mechanism for capacity loss when using NBH is not interlayer formation but rather mechanical pulverization of the Sn electrode due to significant volume change. The Coulombic efficiency data reaches 99% at the second cycle, which indicates a stable anode–electrolyte interface after the first cycle. After solving the interface challenges facing Na-ASSBs, by utilizing novel borohydride electrolytes, attention can begin to focus on remaining challenges such as mechanical degradation of the anode, moving the field one step closer to practical sodium-based energy storage systems.

## Conclusions

Here, we characterize the reduction stability of Na solid electrolytes belonging to three different material families: NYZC, NPS, and NBH. The cells using these electrolytes exhibited different interlayer thicknesses and cycling performance. During electrochemical cycling, Na_2.25_Y_0.25_Zr_0.75_Cl_6_ is reduced to form an electronically conductive and ionically insulating interphase, which propagates rapidly, accelerating capacity loss and increasing cell impedance. The reduction of Na_3_PS_4_ results in the formation of Na_2_S and Na_3_P products, which is a mixed-conducting interphase, that may continue to grow after each cycle. Nonetheless, the growth of the NPS MCI layer thickness and cell impedance was less severe compared to Na_2.25_Y_0.25_Zr_0.75_Cl_6_. Furthermore, no reduction of Na_2_(B_10_H_10_)_0.5_(B_12_H_12_)_0.5_ could be observed under the conditions evaluated here. Cells with Na_2_(B_10_H_10_)_0.5_(B_12_H_12_)_0.5_ exhibited the highest first Coulombic efficiencies and capacity retentions, owing to the superior stability of the NBH electrolyte. This study shows the need to move beyond commonly used electrolytes like Na_3_PS_4_ and to develop new materials that can minimize Na inventory loss and impedance growth to enable practical full-cell architectures.

## Methods

Due to the sensitivity of many solid electrolyte and electrode materials to H_2_O and O_2_ in air, all experiments were performed inside an Ar-filled glovebox, unless otherwise noted.

### Solid-State-Electrolyte Syntheses

Na_2.25_Y_0.25_Zr_0.75_Cl_6_ was synthesized by ball milling a stoichiometric ratio of NaCl (99%, Sigma-Aldrich), YCl_3_ (99.99%, Sigma-Aldrich), and ZrCl_4_ (99.99%, Sigma-Aldrich) according to a previously established procedure.^22^ Na_3_PS_4_ was obtained from a stoichiometric ratio of Na_2_S (99%, Nagao) and P_2_S_5_ (99%, Sigma-Aldrich), as described in ref (49). Na_2_(B_10_H_10_)_0.5_(B_12_H_12_)_0.5_ was synthesized by ball milling a stoichiometric ratio of Na_2_B_10_H_10_ (98%, Katchem) and Na_2_B_12_H_12_ (99.5%, Katchem), which both had previously been dried under vacuum at 175 °C for 48 h. The as-obtained powder material was then again heated under vacuum at 175 °C for 48 h.

Ionic conductivity measurements were performed to determine the ionic conductivity of the obtained electrolyte samples (Figure S6). The ionic conductivities of NYZC, NPS, and NBH were 0.0627, 0.185, and 1.8 mS cm^–1^, respectively.

### Synthesis of NaCrO_2_ and Na_9_Sn_4_ Electrode Materials

NaCrO_2_ (NCO) was synthesized from a stoichiometric ratio of Cr_2_O_3_ (99.97%, Alfa Aesar) and Na_2_CO_3_ (99.5%, Alfa Aesar). The mixture was pelletized and then calcinated under Ar at 900 °C for 10 h before being naturally cooled to room temperature. Na_9_Sn_4_ was synthesized by ball milling Na metal (99.9%) and Sn powder (99%, 10 μm, Sigma-Aldrich) in a 2.25/1 (Na/Sn) ratio. Rietveld refinement confirmed that NCO and Na_9_Sn_4_ were obtained as pure phase (Figure S7).

### Preparation of Sn Electrodes

Sn electrodes were prepared by casting Sn slurry, containing Sn and polyvinylidene fluoride (PVDF) in a 99.5/0.5 weight ratio dispersed in *N*-methyl-2-pyrrolidinone (NMP) solvent, on Al foil. The slurry was dried overnight in a vacuum oven at 80 °C.

### Cell Fabrication

Solid-state cells were assembled in a 10 mm polyether ether ketone (PEEK) die with two Ti plungers. 70 mg of the SSE (NYZC, NPS, NBH) was pressed under 370 MPa to form a rigid SSE pellet. The thickness of the resulting SSE separator layers was ∼500 μm. The Sn anode and the cathode composite containing NaCrO_2_, NYZC, and vapor-grown carbon fibers (VGCF) in an 11/16/1 weight ratio were added to opposing sides of the SSE pellet. 12 mg of cathode composite was used in all experiments. A thin layer of 25 mg of NYZC was added before the addition of cathode composite to ensure that the cathode–SSE interface in all the cells was the same; the difference in cell performance can then be attributed to the anode–SSE interface instability. The architecture of full cells is referred to as Sn | SSE | NYZC | cathode composite (Figure 1). The assembled cells were pressed again at 370 MPa before electrochemical testing. Similarly, Na_9_Sn_4_ | SSE | Sn half cells were assembled to further investigate the anode–SSE interface in later experiments.

### Electrochemical Testing

Galvanostatic cycling of the full cells was performed between 1.7 and 3.4 V at a current density of 0.064 mA cm^–2^. Cycling of the half cells was performed by sodiating the Sn anode until 95% of its theoretical capacity was reached, using a capacity cutoff. The Sn anode was subsequently desodiated until a 2.0 V cutoff was reached, where the applied current density was 0.16 mA cm^–2^.

EIS measurements were performed using a Solartron 1260 impedance analyzer. Impedance measurements were collected using an applied AC amplitude of 30 mV over a frequency range of 1 MHz to 1 Hz. Direct current (DC) polarization measurements were collected using the potentiostat of the same instrument by applying 50 mV and measuring the current response over time. The steady-state current was used to calculate the electronic conductivity values.

### Focused Ion Beam Scanning Electron Microscopy

The cells were extracted from the PEEK dies by removing the Ti plungers and inserting a 10 mm metal rod into the Na_9_Sn_4_ side of the cell. The stacked cell and metal rod were then pressed very slowly to push the pellet out of the top of the PEEK die. The pellet was then mounted onto a carbon tape-covered stub. The sample was then mounted onto an airtight transfer arm while inside the glovebox and sealed. The sample was then transferred into a FEI Scios Dualbeam (Thermo Fisher Scientific) chamber without any air exposure during the transfer. A Ga^+^ source was used for ion-beam milling at 30 kV and 63 nA. Afterward, the sample cross section was cleaned with Ga^+^ at 30 kV and 15 nA. All the imaging was performed with the electron beam source at 5 kV and 0.1 nA. EDS mapping was collected using a 10 keV electron beam with a current of 0.1 nA.

### X-ray Photoelectron Spectroscopy

XPS measurements were performed using a Kratos Axis Supra XPS instrument. Al Kα radiation was used, and the chamber pressure was less than 5 × 10^–8^ torr during operation. A charge neutralizer was used for insulating samples, and the scan resolution was 0.1 eV with a dwell time of 100 ms. CasaXPS was used for data analysis.^50^ The data was calibrated based on the C 1s peak at 285 eV, and a Shirley-type background was used.

### X-ray Diffraction

XRD measurements were performed using a Bruker APEX II Ultra diffractometer with Mo Kα (λ = 0.7093 Å) radiation at 40 kV and 40 mA or a Bruker 3 circle diffractometer with Cu Kα (λ = 1.5406 Å) radiation at 45 kV and 50 mA on flame-sealed boron-rich glass capillaries in a Debye–Scherrer geometry. The diffraction images gathered by the 2D detector within an angular range of 5–40° for the Mo source and a range of 10–90° for the Cu source were merged and integrated with DIFFRAC.EVA (Bruker, 2018) to produce 2D plots. Rietveld refinement was performed using the FullProf software suite.^51^

## Supporting Information Available

Electrolyte linear sweep voltammetry measurements, XRD data for electrolyte/Sn mixtures, additional EDS mapping data for the tin–electrolyte interfaces, symmetric cell data, long cycling failure analysis, electrolyte conductivity measurements, and Rietveld refinement analysis for NaCrO_2_ and Na_9_Sn_4_ phases (PDF)
