# Supplementary material for: Evaluating Electrolyte–Anode Interface Stability in Sodium All-Solid-State Batteries
Source: ACS Appl Mater Interfaces. 2022 Oct 14;14(42):47706–15. doi: 10.1021/acsami.2c12759 (PMC9614718; doi:10.1021/acsami.2c12759)
Supplement: Supplementary file 1 — am2c12759_si_001.pdf [file am2c12759_si_001.pdf]

Supporting Information

Evaluating Electrolyte−Anode Interface Stability in Sodium All-Solid-State Batteries

Grayson Deysher[a], Yu-Ting Chen[a], Baharak Sayahpour[a], Sharon Wan-Hsuan Lin[b], So-Yeon Ham[a], Phillip Ridley[b], Ashley Cronk[a], Erik A. Wu[b], Darren H. S. Tan[b], Jean-Marie Doux[b], Jin An Sam Oh[b], Jihyun Jang[b], Long Hoang Bao Nguyen[b], Ying Shirley Meng[b,c,*]

[a] Program of Materials Science and Engineering, University of California San Diego, La Jolla, CA 92093, United States.

[b] Department of NanoEngineering, University of California San Diego, La Jolla, CA 92093, United States.

[c] Pritzker School of Molecular Engineering, The University of Chicago, Chicago, IL 60637, United States

* Corresponding author: shirleymeng@uchicago.edu

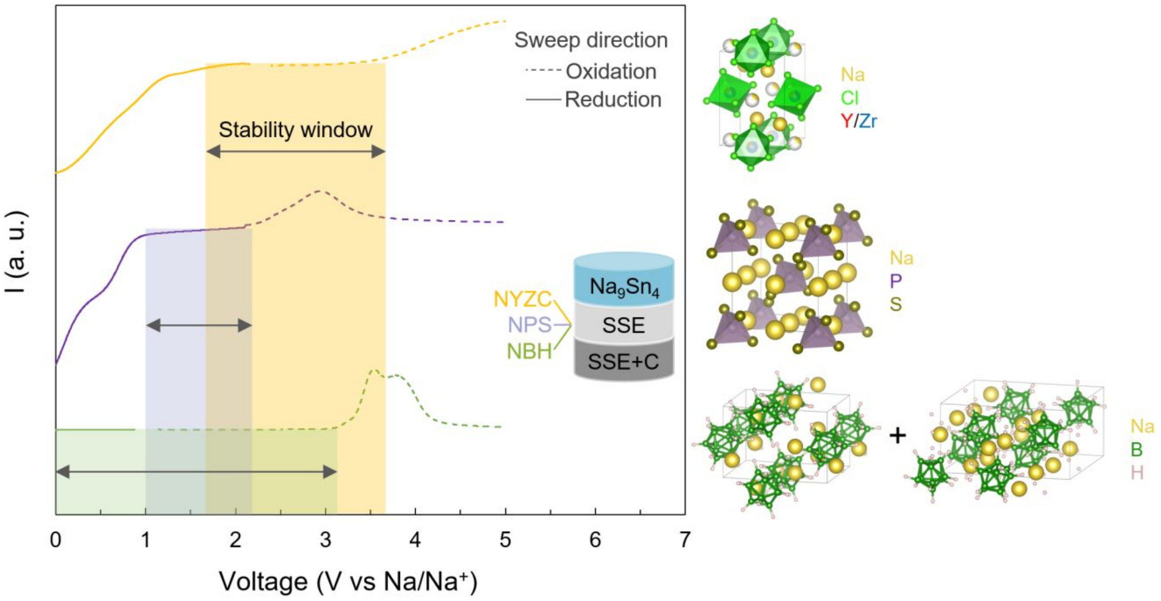

Figure S1. Linear sweep voltammograms for NYZC, NPS, and NBH.

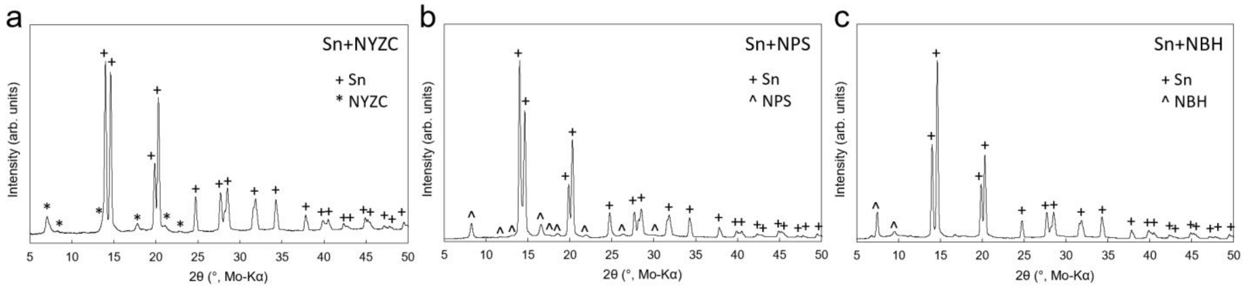

Figure S2. XRD patterns for mixtures of Sn with a) NYZC, b) NPS, and c) NBH after heating at 80 °C for 10 h.

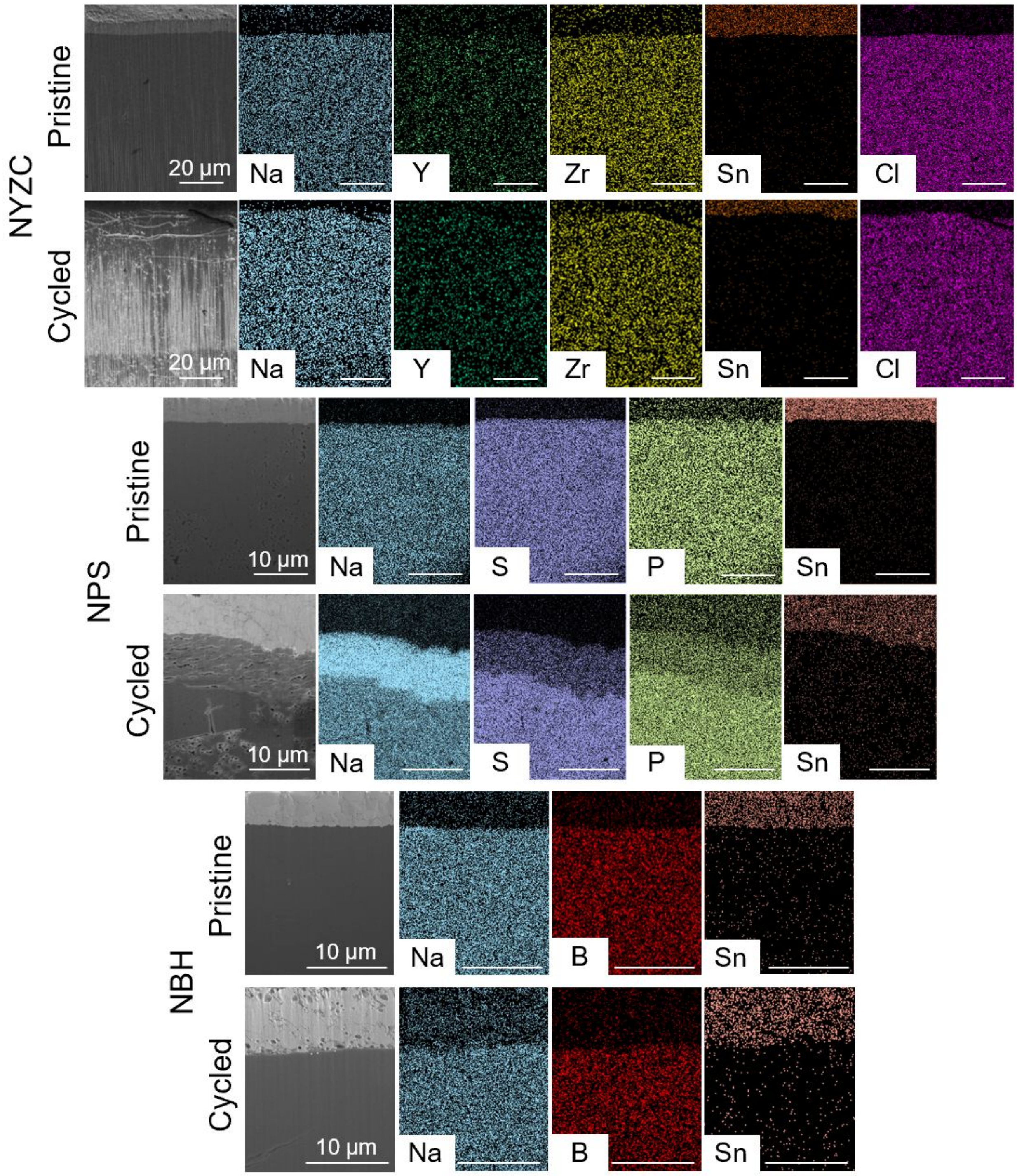

Figure S3. FIB-SEM with EDS mapping of Sn | SSE | Na_9Sn_4 cells.

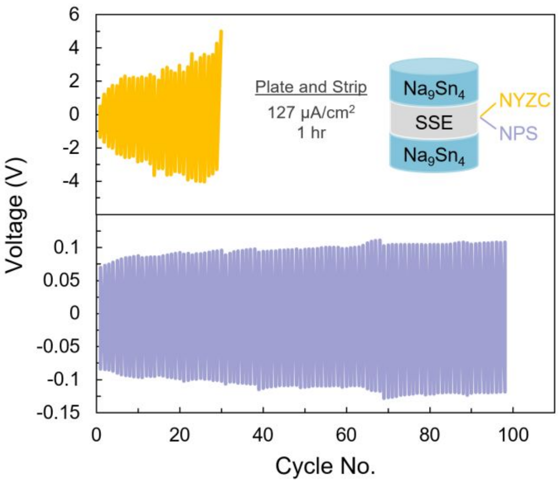

Figure S4. Symmetric cell evaluation using NYZC and NPS electrolytes.

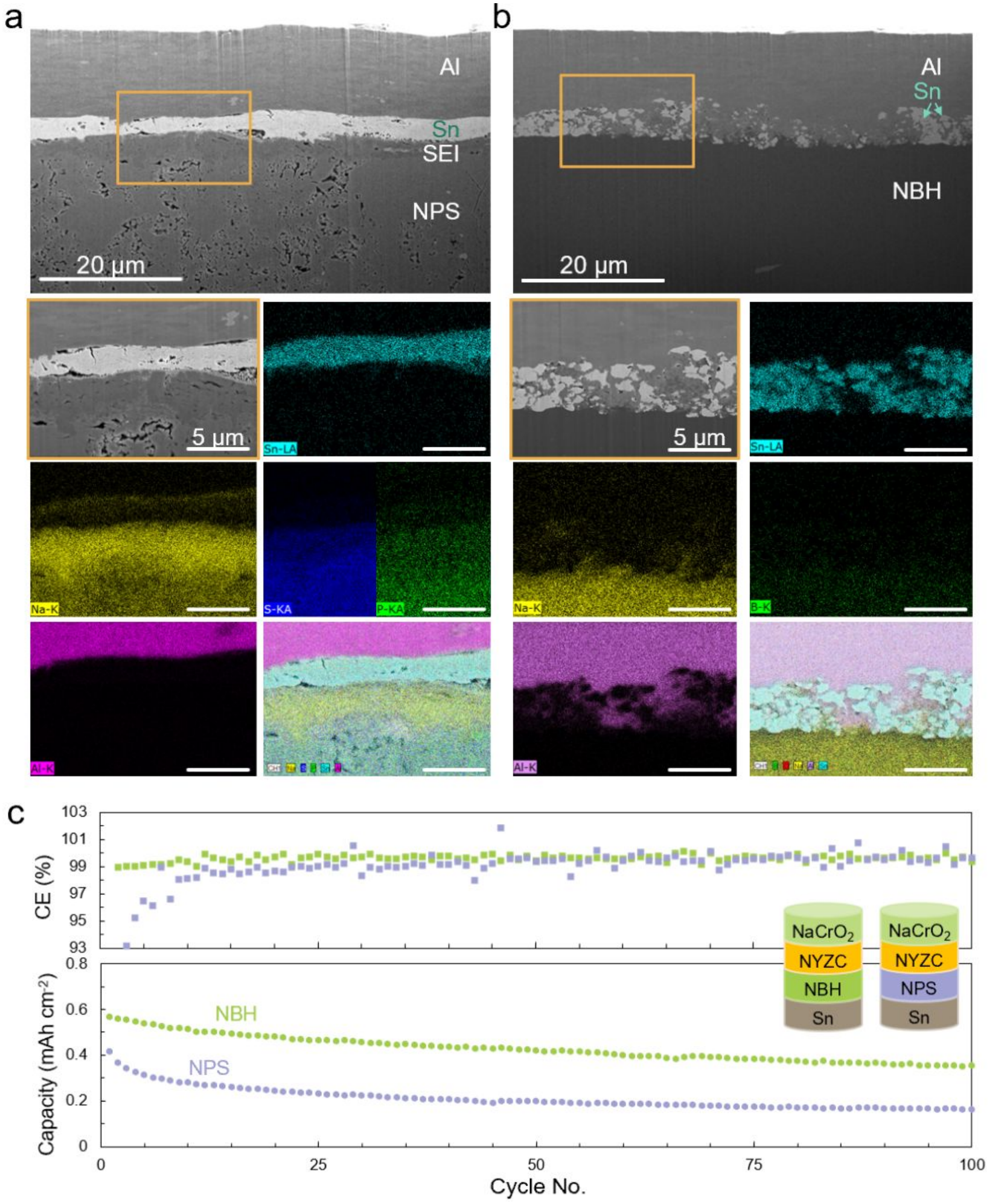

Figure S5. FIB-SEM anode cross-section and EDS elemental mapping for (a) Sn | NPS | NYZC | $NaCrO_2$ and (b) Sn | NBH | NYZC | $NaCrO_2$ cells after cycling. (c) Electrochemical cycling data for both cells.

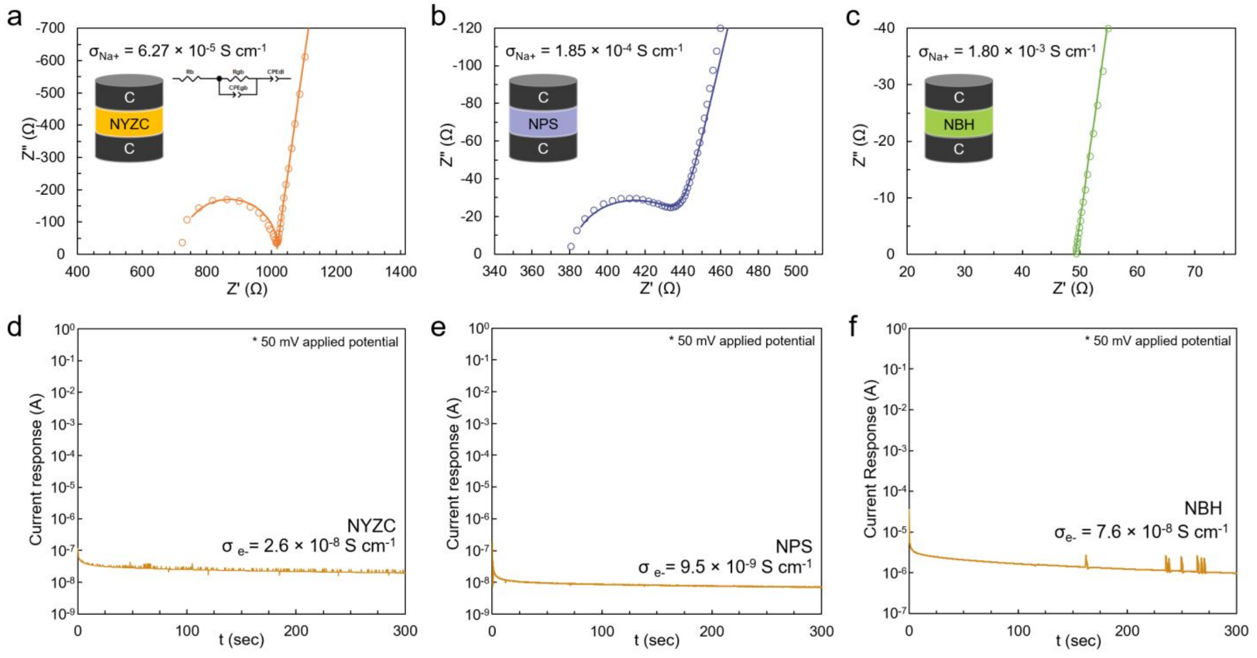

Figure S6. EIS measurements of (a) NYZC, (b) NPS, and (c) NBH showing Na^+ conductivities of 0.0627, 0.185, and 1.8 mS/cm respectively. DC Polarization measurements of (d) NYZC, (e) NPS, and (f) NBH showing e^- conductivities of 26, 10, and 76 nS/cm respectively.

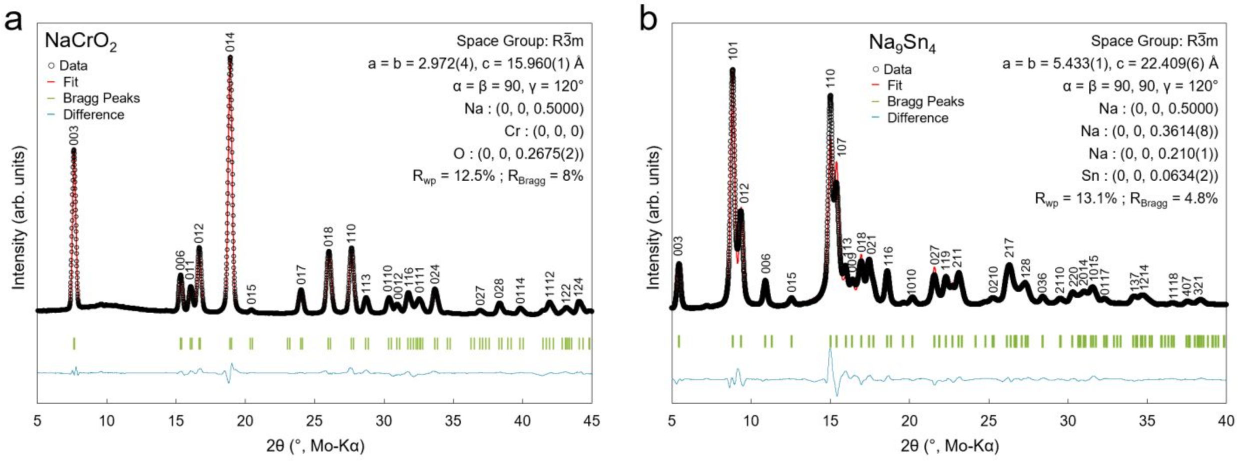

Figure S7. XRD refinement results of (a) $NaCrO_2$ and (b) Na_9Sn_4.
